# A systems approach to developing user requirements for increased pulmonary rehabilitation uptake by COPD patients

**DOI:** 10.1038/s41533-024-00370-1

**Published:** 2024-07-16

**Authors:** Frances Early, James Ward, Alexander Komashie, Timoleon Kipouros, John Clarkson, Jonathan Fuld

**Affiliations:** 1 Cambridge University Hospitals NHS Foundation Trust, Cambridge, UK; 2 Department of Engineering, University of Cambridge, Cambridge, UK; 3 The Healthcare Improvement Studies (THIS) Institute, University of Cambridge, Cambridge, UK

**Keywords:** Rehabilitation, Chronic obstructive pulmonary disease

## Abstract

Chronic Obstructive Pulmonary Disease is a progressive lung disease associated with anxiety, depression, and reduced health-related quality of life. Pulmonary rehabilitation (PR) is a cost-effective and transformative treatment, but 31% of referred patients do not take up their PR appointment. The study aimed to develop user requirements for an intervention to increase PR uptake. A systems approach, the Engineering Better Care framework, was used to develop a system map of the PR pathway, translate evidence-based user needs into user requirements, and validate the user requirements in a stakeholder workshop. Eight user requirements addressed patient and health care practitioner needs to understand what PR entails, understand the benefits of PR and have positive conversations about PR to address patient concerns. The solution-independent user requirements can be applied to the development of any intervention sharing similar goals. The study demonstrates potential in taking a systems approach to more challenges within respiratory medicine.

## Introduction

Chronic Obstructive Pulmonary Disease (COPD) is a progressive lung disease characterised by breathlessness and persistent cough. Patients report high symptom burden with associated anxiety and depression, reduced quality of life and loss of independence and freedom in an ever-shrinking world^1–3^. Pulmonary rehabilitation (PR) is a “…comprehensive intervention based on a thorough patient assessment followed by patient-tailored therapies that include, but are not limited to, exercise training, education, and behaviour change, designed to improve the physical and psychological condition of people with chronic respiratory disease and to promote the long-term adherence to health-enhancing behaviours.”^4^ It is a cost-effective and transformative treatment for COPD^5^. PR reduces hospital admissions and leads to clinically significant improvements in breathlessness, fitness and quality of life^6,7^. Referrals are mainly from primary care or from the community for patients with stable COPD (66.8%); 5.2% of referrals follow hospital admission for an acute exacerbation of COPD^8^.

In England, around 31% of referred COPD patients do not take up their PR appointment, more so from deprived communities^9,10^. Systematic reviews have found insufficient high-quality evidence to make clear recommendations for effective interventions to increase uptake^11,12^. In a previous study, we identified barriers and enablers to PR uptake at several stages on the PR pathway within and between primary care, PR providers and patients. In patient encounters with primary care, individual barriers to uptake, such as lack of confidence to exercise or not believing that PR will help, were often not addressed effectively. Patients needed a better understanding of how PR could benefit them personally and better support around emotional or practical challenges they faced in attending PR. Meeting such needs was a challenge for many primary care clinicians. There was an appetite for interventions that could address these needs^13^.

Facilitating the uptake of PR from primary care while meeting the needs of a wide range of users is a system-wide challenge involving patients, carers, GPs, practice nurses, physiotherapists who deliver PR and healthcare commissioners. Meeting the challenge requires a systems approach to take into account the needs of different users and the constraints imposed by complex supporting systems^14^. There is evidence for the success of systems approaches in healthcare, but they are not well established in healthcare intervention design^15^. The Engineering Better Care (EBC) framework for developing and improving healthcare settings is built on systems engineering principles that emphasise a holistic approach to design and are translated for healthcare applications^16^. Coproduced by NHS clinicians, managers, policy makers, patients and systems engineers, its development was led by the Royal Academy of Engineering in partnership with the Royal College of Physicians and the Academy of Medical Sciences. EBC is rooted in an established engineering model for the design and delivery of safe and successful systems. EBC principles have been refined into a how-to guide – the Improving Improvement Toolkit (http://www.iitoolkit.com). The Toolkit has been used to apply a systems approach to the design of a range of healthcare delivery challenges across the NHS and policy design across multiple areas of the UK Government.

The aim of the current study was to use the EBC framework to develop a set of user requirements for an intervention that could be delivered in primary care to increase uptake to PR.

## Methods

### Study design

**Fig. 1:**
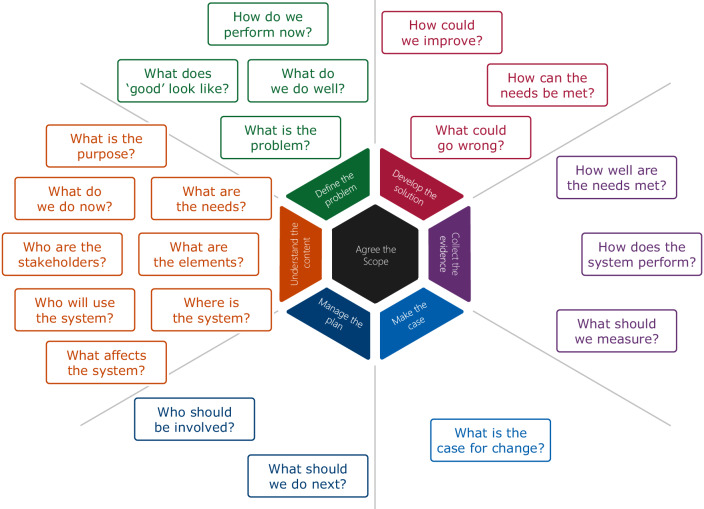
The Hexagon Improvement Model. The hexagon at the centre of the model shows the seven activity strands that are present throughout the Engineering Better Care improvement process. In the early stages, Understand the Context and Define the Problem are given most emphasis and activities continue in a clockwise direction. The Engineering Better Care process also defines questions that are key to the systems approach. The questions are shown in the boxes mapped to the seven activity strands but the mapping need not be definitive.

The Engineering Better Care (EBC) framework incorporates strands of activity. These are illustrated in the Hexagon Improvement Model (Fig. 1). *Agree the Scope*, at the centre of the hexagon, underpins six outer activities. The six outer activities begin with *Understand the Context* followed by *Define the Problem* and so on in a clockwise direction. As new data are generated by each activity, outputs from previous activities may be refined. Within each activity, specific questions help drive exploration. Figure 1 shows the questions roughly mapped to the activities but this mapping is not definitive. The Improving Improvement Toolkit provides tools and techniques to help answer the questions. The questions are optional and only need to be asked when helpful, hence we chose a subset.

The current study took place at an early stage in the design process—the development of user requirements—and so focused on the first two activities (*Understand the Context* and *Define the Problem*) and on the questions: *“Where is the system?” “‘What are the elements?” “‘Who are the stakeholders?” “‘Who will use the system?” “What affects the system?” “What are the needs?” “What does good look like?”*

This work was part of a broader mixed methods package of work that received a favourable opinion from the Cambridge Central Research Ethics Committee (REC reference number 17/EE/0136), was granted approval by the Health Research Authority and was registered with the ISRCTN registry (trial ID: ISRCTN20669629, assignment date 20 March 2018, trial start date 1 April 2016)^17^.

**Fig. 2:**
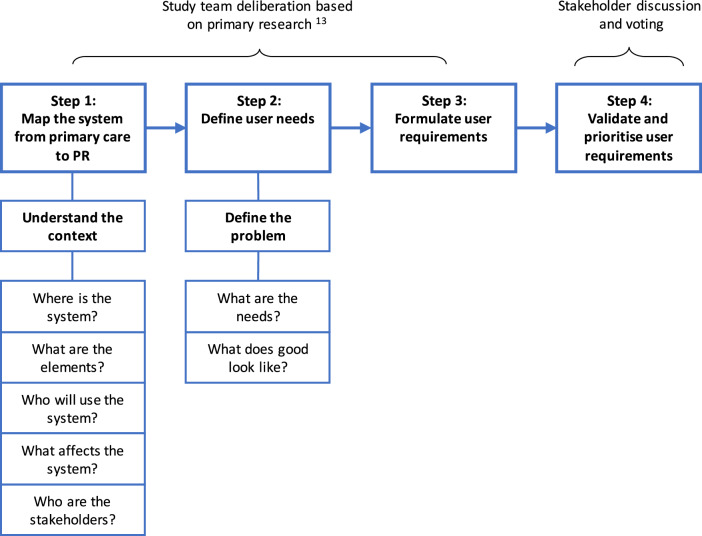
The development of user requirements. Steps 1, 2 and 3 were undertaken by the study team prior to stakeholder validation in step 4.

Four steps were followed to develop the user requirements (Fig. 2).

### Step 1: Map the system

The expert study team (respiratory clinicians, systems engineers and applied health researchers) developed a system map of a typical pathway from primary care to PR to identify associated stakeholders and users and the system within which the pathway sits. They defined which areas of the pathway were in-scope for the intervention, i.e. where an intervention to increase uptake could be delivered in a primary care setting. In order to map and understand the system environment they drew on barriers and enablers to uptake previously identified by the team^13^, questions in the Hexagon Improvement Model (*‘Where is the system?’ ‘What are the elements?’ ‘Who are the stakeholders?’ ‘Who will use the system?’ ‘What affects the system?’*) and tools from the Improving Improvement Toolkit (flowcharting, storyboarding and expert reviews).

### Step 2: Define user needs

The EBC approach describes user needs as things that users believe they must have. We distinguish these from user requirements, which are things that the solution (i.e. the intervention) must have in order to satisfy the user needs.

To answer the question *‘What are the needs?’* the study team mapped the barriers and enablers generated by stakeholders in their previous work to the system map, identifying those that were in-scope for an intervention to address. This previous work included patients who had declined PR, those who had never been referred to PR and patients who had attended PR^13^. In-scope barriers and enablers were those that a) mapped to areas of the pathway that were in-scope for the intervention and b) related to generic rather than local factors (focusing on generic factors would facilitate intervention rollout). Consensus was achieved through deliberative discussion. A further deliberative process was used to theme the in-scope barriers and enablers in order to define the user needs.

### Step 3: Formulate user requirements

User requirements address the question *‘What does good look like?’* The study team used a deliberative process to formulate actionable user requirements that would answer this question and satisfy the user needs. While it would have been possible to create many user requirements at a fine level of detail, we required a number that was manageable for stakeholders to review in a workshop. This therefore determined their level of detail. For each user requirement, the team developed an evidence-based supporting statement that drew on the underpinning data in Early et al^13^. Each statement described a) the user needs addressed by the requirement; b) examples of related problems identified in the data and c) examples of what ‘good’ would look like if the user requirement was satisfied. The wording was informed by guidance on how to create good requirements^18^. For example, they should be clear and concise and should not overly constrain the design by specifying a solution, rather they should express an intervention end-goal that can be evaluated.

Step 1 and Step 2 were completed over 12 hours in four research team meetings (each with 3-5 team members present) held between October and November 2018.

### Step 4: Validate and prioritise user requirements

User requirements and supporting statements were presented to stakeholders in a one-day workshop in November 2018. Users and providers of the system (people living with COPD and their carers/families, practice nurses, GPs, PR providers) and other stakeholders (commissioners, NHS England) were eligible to take part. Purposive and snowball sampling was used to recruit participants with knowledge and/or experience of the PR pathway. All participants gave written consent.

The aim of the workshop was for stakeholders to understand the user requirements and for the team to assess the variation in opinions about whether each requirement was ‘Essential’ or ‘Desirable’. A ‘not required’ option was not included because the requirements were derived from research data that represented known aspects of the system.

The workshop progressed as follows:

1. Throughout the workshop, we aimed to promote an inclusive culture through a welcoming and encouraging tone. All participants introduced themselves at the start. The centrality of the patient voice was reinforced by beginning the workshop with a patient presenting his own story about his experiences with COPD and PR. Following this, the researchers presented two anonymised patient stories based on accounts from their previous research – one of a patient who had declined PR and one of a patient who had not been referred to PR – and a short vignette of an anonymised PR provider perspective of the patient experience at PR^13^.
2. The workshop process was explained to the participants and a practice exercise conducted with a non-clinical example.
3. Participants were divided into three mixed groups of 5-6 comprising patients, carers, clinicians and other stakeholders. Each group sat at a separate table with a facilitator from the study team. Groups were given A4 paper copies of the user requirements and supporting statements (Supplementary Notes 1). Each person had two A5 sized voting cards, one labelled ‘Essential’ and one labelled ‘Desirable’.
4. Each user requirement, with supporting statement, was presented in turn verbally to the whole workshop. After each requirement, participants voted in their groups on whether it was ‘Essential’ or ‘Desirable’ by each person simultaneously holding up one of their voting cards. The facilitator recorded the results by hand in a matrix. The group then discussed the requirement for three minutes. Facilitators ensured that the patient and carer participants were able to contribute their views by inviting contributions from any participants who did not freely give a view. Participants then voted a second time in the same manner, the facilitator again recorded the results by hand in the matrix. This activity was a hybrid between nominal group technique, in which individuals work independently in the presence of one another, and a focus group technique to encourage discussion^19–21^ hence participants voted twice – once independently and once after group discussion.
5. Facilitators aggregated the votes from all the tables to produce an aggregated score for each user requirement as follows:
  $$[(3* {\rm{TE}}1){\mbox{-}}(2* {\rm{TD}}1)]+[(6* {\rm{TE}}2){\mbox{-}}1* {\rm{TD}}2\left.\right)]$$
  TE1 stands for Total Essential votes at 1st voting, TD1 stands for Total Desirables at 1st voting, TE2 stands for Total Essentials at 2nd voting and TD2 stands for Total Desirables at 2nd voting. In both rounds, more weighting was given to Essentials versus Desirables, as reflected in the constants chosen (3, 2, 6 and 1). In the second round, even greater weighting was given to Essentials over Desirables. This recognised the importance of the discussions in between the two voting rounds. The constant values were adjusted until the graphs appeared meaningful when applied to dummy data, i.e. accentuated the differences in importance between the requirements. Following this adjustment, the aggregated score value of each requirement ranged from 0 to 180.
6. Results were presented to the workshop for verbal confirmation and questions.

### Reporting summary

Further information on research design is available in the Nature Research Reporting Summary linked to this article.

## Results

### System map

**Fig. 3:**
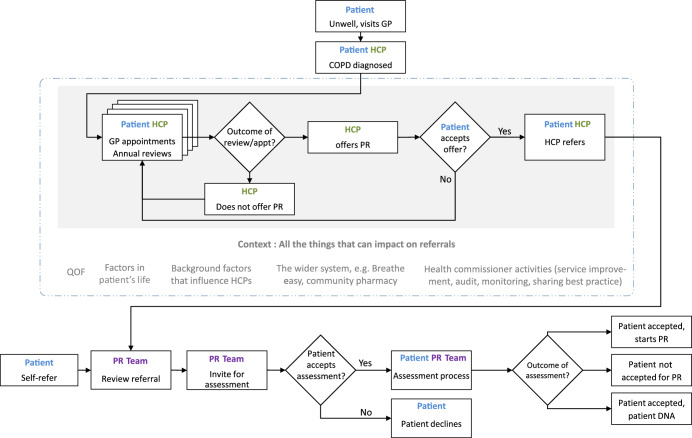
System map. A typical referral pathway from primary care to pulmonary rehabilitation. The area that is in scope for the study is shaded in grey.

Figure 3 shows the system map, illustrating a typical pathway from primary care to PR. The pathway begins with the patient’s symptom experience and diagnosis, followed by planned and ad hoc encounters with primary care. When a PR referral is made, the PR service contacts the patient to make an assessment appointment. If the patient attends the assessment, they are invited to begin the programme or excluded as not eligible. Not all local pathways are identical but our system map incorporates key generic elements that are likely to be represented in some way in most pathways. The shaded area is in-scope for this study, i.e. the area where an intervention to increase uptake could be delivered in a primary care setting.

### User needs

Of the barriers and enablers to uptake we previously identified, twelve barriers and eight enablers were identified as in-scope for the intervention (Table 1)^13^. Three categories of user needs were derived from these: 1) Patients and clinicians needed to know and understand what happened on a PR programme; 2) Patients and clinicians needed to understand the benefits of PR as a treatment and how an individual patient could personally benefit; 3) Patients needed to be reassured about anxieties or concerns and feel positive about attending PR.

**Table 1:** In scope barriers and enablers identified by Early et al.^13^ translated into user needs and user requirement statements.

Barriers that prevent uptake	Enablers that support uptake	Categories of user needs	Actionable user requirements
Patient has little knowledge of PR. HCP lacks appropriate training and education about PR. Medical terminology that the patient cannot understand, e.g. ‘pulmonary’, ‘rehabilitation’.	Patients can see, hear or read testimonies from others who have attended PR.	UNDERSTANDING WHAT PR ENTAILS Patients and clinicians need to know and understand what happens on a PR programme.	Help the patient understand what happens on a PR programme. Help the HCP to understand what happens on a PR programme.
Patient believes that PR is pointless. Patient believes they are coping well and sees no need for PR. Patient is satisfied with care and sees no need for PR. HCP finds it difficult to connect PR benefits to patient needs. HCP finds it difficult to ‘light the spark’.	Patient believes that PR will help them. Patient is struggling to cope with their COPD. HCP values PR as a treatment for COPD.	UNDERSTANDING THE BENEFITS OF PR Patient needs to understand how they will be able to benefit personally from PR. Patients and clinicians need to understand and value the role of PR in COPD treatment.	Help the patient understand how they will benefit personally from PR. Help the HCP to understand the benefits that patients gain from PR. Help the HCP to feel positive about PR and value it as a treatment.
Patient believes that exercise is impossible for them. Patient receives referral late in the disease course when they feel less able to exercise. Patient is anxious about joining a group. Patient fears being pressured to quit smoking.	Patient believes that exercise is possible for them. Patient wants support for managing their COPD. HCP reassures the patient about attending PR. HCP emphasises to the patient what support will be available at PR.	FEELING POSITIVE ABOUT ATTENDING PR Patient needs to have positive feelings about attending PR. Patient needs reassurance about attending PR.	Help the patient to feel positive about attending a PR programme. Help the patient to feel reassured about any anxieties they have about PR. Help HCPs, patients and carers to feel they have had a positive conversation about PR.

Barriers and enablers that were out of scope related to patient-specific circumstances, primary care organisational factors, practical issues outside of primary care control (e.g., transport, health service organisation and delivery) and lack of clinician training in specialist respiratory skills (Supplementary Notes 2).

### User requirements

Eight actionable user requirements were formulated from the user needs:

Requirement 1: Help the patient understand what happens on a PR programme

Requirement 2: Help the patient to feel positive about attending a PR programme

Requirement 3: Help the patient to understand how they will benefit personally from PR

Requirement 4: Help the patient to feel reassured about any anxieties they have about PR

Requirement 5: Help the HCP to understand what happens on a PR programme

Requirement 6: Help the HCP to understand the benefits that patients gain from PR

Requirement 7: Help the HCP to feel positive about PR and value it as a treatment

Requirement 8: Help HCPs, patients and carers to feel they have had a positive conversation about PR

The evidence-based supporting statements for the requirements are shown in Supplementary Notes 1.

### Validation and prioritisation of user requirements

Eighteen participants (15 female, 3 male) took part in a stakeholder workshop: 5 patients (4 female, 1 male), one carer (female), 6 physiotherapists from PR providers, 3 GPs, one practice nurse, one commissioner of PR services and one representative from the British Lung Foundation.

**Fig. 4:**
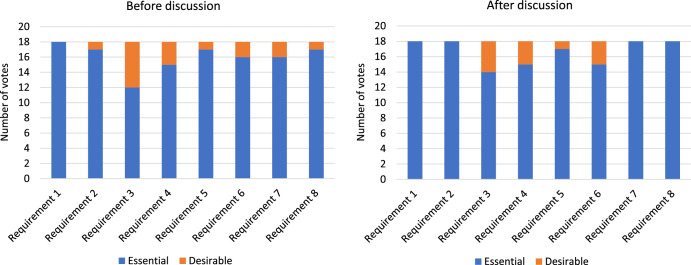
User requirement voting. The number of votes for ‘Essential’ and ‘Desirable’ per user requirement before discussion and after discussion.

Figure 4 shows the number of votes cast for ‘Essential’ and ‘Desirable’ for each user requirement before and after group discussion. User requirement 1 (help the patient understand what happens on a PR programme) was voted ‘Essential’ before and after discussion by all participants. For other requirements, the number of votes for ‘Essential’ or ‘Desirable’ changed slightly before and after discussion. A breakdown of raw scores is shown in Supplementary Table 1 and Supplementary Table 2.

**Fig. 5:**
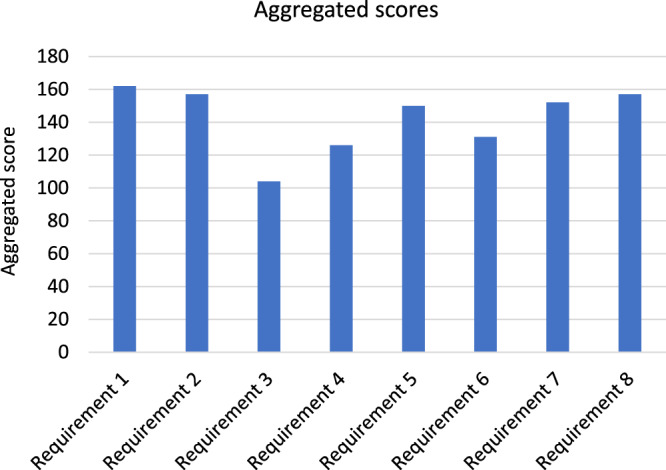
User requirement aggregated scores. The aggregated score for each requirement formulated from a weighting of before and after votes.

Figure 5 shows aggregated scores for each user requirement after the two rounds of voting. Specific values are shown in Supplementary Table 3. The numerical values from 0-180 are arbitrary and have no objective meaning; their value is to provide a consistent way of measuring participants’ responses. The user requirements were ranked in importance as follows:

Requirement 1: Help the patient understand what happens on a PR programme

Requirement 2: Help the patient to feel positive about attending a PR programme

Requirement 8: Help HCPs, patients and carers to feel they have had a positive conversation about PR

Requirement 7: Help the HCP to feel positive about PR and value it as a treatment

Requirement 5: Help the HCP to understand what happens on a PR programme

Requirement 6: Help the HCP to understand the benefits that patients gain from PR

Requirement 4: Help the patient to feel reassured about any anxieties they have about PR

Requirement 3: Help the patient to understand how they will benefit personally from PR

While requirements 1, 2 and 8 ranked the highest, overall there was little to distinguish between the importance of the eight user requirements.

## Discussion

This study demonstrates how a systems approach was used to develop eight user requirements for an intervention to increase PR uptake that could be delivered in primary care and address identified needs of patients and clinicians. The user requirements are mutually supportive, for example a good understanding of PR by a clinician will enable them to better allay patient concerns about attending PR and link potential benefit to the patient’s own circumstances. The user requirements are evidence-based, derived from barriers and enablers to PR uptake identified in prior research^13^, and are solution-independent, so can inform the development of a range of interventions in primary care settings.

Engineers have long recognised the tendency to rush into ‘cutting metal’ early in the mistaken belief that this will advance a project. This may provide a comforting illusion that something useful is being done but avoidance of a detailed understanding of the problem and clear statements of requirements is known to lead to serious consequences later in the life of a system, product, or intervention^22^. While the subject of requirements is well established in fields such as engineering design, systems engineering and software engineering, it is new to healthcare research. Of the limited examples in healthcare, information technology appears the most common area^23–25^. An example in primary care investigated requirement capture issues in the design of primary health facilities in the United Kingdom^26^. The study found poor management of design because of inappropriate processes with inconsistent requirements management. In contrast, clear and consistent requirements ensure that the right intervention is designed and can be appropriately tested. Requirements have played a crucial role in the design and delivery of successful projects in systems and software engineering^27^.

Requirements emerge from a clear understanding of user needs, and state in clear terms what a system or intervention should do in order to meet those needs. The process has been defined as: “… the iterative process by which the needs, preferences and requirements of individuals and groups – stakeholders – significant to the product development are researched and identified”; overall, requirements capture defines customers’, users’ and market requirements, design requirements and technical requirements^28^. Developing requirements can therefore be an extensive task as is the case in systems engineering^22,29^. It is important that efforts invested in developing requirements are appropriate to the scale of the system or proposed intervention^30^. In systems and software engineering, requirements are treated to an extent that is beyond the scope of the current paper. The few examples in healthcare are limited in scope; however, some highlight the variety of ways that requirements can be fully explored. For example, Zillner et al. studied user needs and requirements analysis for big data applications in healthcare. They distinguished between requirements that were business-related, technical related and those that were both business and technical related^25^.

The more comprehensive the requirements are, then the more fully the system or intervention is defined, and the chances of success improved. However, Davis has argued for what may be described as context-specific requirements by stressing the idea of “just enough” requirements^30^. In line with Davis, we consider the findings reported in this study a sufficient starting point in requirement development in a field for which the concept is new. The user requirements developed here were derived from clinicians’ and patients’ needs to understand PR and its benefits and to address patient anxiety. These factors have been frequently identified as important barriers to PR uptake. The need for interventions targeting these areas has been highlighted elsewhere^31^.

While Requirement 1 (help the patient understand what happens on a PR programme), requirement 2 (help the patient feel positive about attending a PR programme) and requirement 8 (help HCPs, patients and carers to feel they have had a positive conversation about PR) were voted as the three most important to prioritise, there was very little difference between the importance of all eight. In cases of intervention design where there is a large number of requirements and several constraints to be met then trade-offs may be needed, and differences in importance could provide a basis for agreeing trade-offs. In our case, there was no evidence from the stakeholder workshop that any of the requirements were rejected by participants or had to be traded-off for any system constraint.

The practical implications of the user requirements are consistent with the conceptual framework of access to healthcare described by Levesque et al. who argue that access to optimal care requires the person to be fully engaged in care^32^. They define access as the opportunity to reach and obtain appropriate services in situations of perceived need for care. This involves expectations, health literacy, knowledge about services and their usefulness, health beliefs and fit between services and patient need. An intervention that met our eight user requirements would address these factors.

The user requirements are solution-independent. They do not specify how an intervention might satisfy the requirements or what any intervention content might be. Rather, they provide intervention developers with a set of validated, evidence-based end goals that could be met through a variety of solutions. Examples are suggested here to illustrate a range of potential approaches. Two user requirements (1 and 5) address patient and clinician needs to understand what happens on a PR programme. Interventions could provide clear, printable information about PR so that clinicians can accurately describe to patients what happens at PR and/or patients could receive first-hand testimonies from previous PR participants about the PR experience. Three user requirements (3, 6 and 7) address patient and clinician needs to understand and value the benefits of PR as a treatment and the ways in which it could personally benefit the patient. An intervention could provide tailored resources that enable the clinician and patient to generate a personalised understanding of why PR is an important part of the patient’s treatment and how it will support them to overcome their particular symptoms and challenges. Three user requirements (2, 4 and 8) address patient and clinician needs for reassurance about patient anxieties and to have a positive conversation about PR. An intervention could provide tailored resources to address the patient’s anxieties so that they feel confident to participate in PR and/or could support clinicians in the skills needed to have sensitive conversations about patient concerns and anxieties.

Strengths of our approach are twofold. First, the EBC framework ensured that the user requirements were underpinned by a rigorous and systematic approach. The likelihood of laying the foundations for interventions that are fit for purpose was improved by ensuring a thorough understanding of the problem and exploring what mattered to a wide range of stakeholders. Second, setting user requirements is an early step in intervention development and the user requirements are solution-independent. By not predetermining the format or content of any subsequent intervention, intervention developers have maximum flexibility to develop a solution that is appropriate for their own context. The user requirements validated here therefore offer a broad contribution to intervention development in this field because they could be applied to the development of a range of interventions that share similar goals.

Developing, specifying and managing requirements is not a simple process and can take up to 30% of the total project time, particularly for complex products that are heavily regulated such as medical devices^33^. A potential limitation of our approach is that the study focused on the early stage of a full design process. Further work will be required to develop a comprehensive requirements list beyond user requirements, e.g. requirements also comprise technical, business and regulatory requirements. Additionally, our stakeholder validation workshop included people living with COPD, their carers/families, practice nurses, GPs and PR providers who were physiotherapists. PR is a multi-disciplinary programme and so inclusion of other clinical stakeholders who contribute to PR (e.g. occupational therapists, dieticians, nutritionists) would have broadened the stakeholder perspective at this stage. However, we were encouraged by the close agreement achieved on the requirements at this stage and other stakeholder perspectives can be incorporated as the design progresses. Lastly, requirements tend to be refined during the design process and this principle will apply to our user requirements. For example, further detail, such as performance limits that form the basis of testing, tends to be added as the design progresses. Similarly, as the form of the solution takes shape, this tends to dictate new requirements and may lead the refinement of existing requirements. For example, new stakeholders may join the design process at later stages and this brings opportunities to ensure the continued validity of the requirements and to make any refinements as required. Thus, our user requirements should be seen as a comprehensive first attempt, to be further developed during the later stages of intervention design.

In conclusion, we have argued that effectively increasing uptake to pulmonary rehabilitation from primary care while meeting the needs of a wide range of stakeholders is a systems challenge. We employed the Engineering Better Care systems approach to address the challenge of developing user requirements for an intervention to support uptake of pulmonary rehabilitation that could be delivered in primary care. We developed eight validated, evidence-based and solution-independent user requirements to address evidence-based user needs. We found few studies on systems approaches to requirements definition in respiratory medicine. This study demonstrates there may be potential in taking a systems approach to more challenges within this discipline.

### Supplementary information

Supplemental material

Reporting summary

## Data Availability

All data generated and analysed during this study are included in this published article and its supplementary information files.
