# Supplementary material for: A systems approach to developing user requirements for increased pulmonary rehabilitation uptake by COPD patients
Source: NPJ Prim Care Respir Med. 2024 Jul 16;34:20. doi: 10.1038/s41533-024-00370-1 (PMC11252258; doi:10.1038/s41533-024-00370-1)
Supplement: Supplementary file 1 — Supplemental material [file 41533_2024_370_MOESM1_ESM.pdf]

Supplementary Notes 1:

Evidence-based supporting text for user requirements

Requirement 1: Help the patient understand what happens on a PR programme

User needs that the user requirement will address

- Newly diagnosed patients need to be aware of PR and its role in treating COPD.
- Patients need to understand what happens at PR and understand that it is not just exercise. Patients and clinicians need engaging educational and motivational materials.
- Paper information is important because some will not go online.

Examples of related problems identified in the primary data

- Patients do not know what PR is or have not heard of it.
- No one has explained it to them.
- Not enough people in the community have done PR and so there is not storytelling in lunch clubs or the community to spread the word.
- The patient does not know anyone who has been to PR.

Examples of what 'good' would look like if the user requirement were satisfied

- Patients are aware of PR even if they do not know about it in detail. Clinicians can build on this awareness when they talk to patients about PR.
- The patient gets good quality information about PR that is easy to understand, covering details of the service and what happens in the class.
- The referrer is sure that they have provided the patient with the information they need to make an informed decision.
- The patient is offered opportunities to find out more about PR to support their decision-making, e.g. being able to visit PR class as an "observer".

Requirement 2: Help patient to feel positive about attending a PR programme

User needs that the user requirement will address

- Patients need information about the benefits of PR and how it has helped others.
- Patients need information that is visually enticing.
- Patients need support to overcome pessimism about their condition.

Examples of related problems identified in the primary data

- The patient believes that nothing will make a difference to their condition. If PR does not cure COPD then it is not worth it.
- It is pointless doing exercises if you cannot walk far.
- The patient is not confident and afraid of new situations. They are embarrassed about their condition.
- They feel guilty about smoking.
- The term 'rehab' is off-putting and associated with blame.
- Patients do not see PR as a lifesaver in the way that a cardiac patient would see cardiac rehab as a lifesaver.
- In deprived areas, patients may face more challenges in looking after their health, which could affect motivation to attend PR.

Examples of what 'good' would look like if the user requirement were satisfied

- Patient testimonies are available from others who have attended PR.
- The GP practice has patient champions who talk to other patients.
- Information is available that appeals to family members/carers (the 'persuaders').

Requirement 3: Help the patient to understand how they will benefit personally from PR

User needs that the user requirement will address

- Information needs to be personalised, relating PR to the individual's problems rather than generic benefit.
- Information needs to give the patient confidence that PR will help them learn what they can do to tackle their problems if they are struggling.
- Patients need to understand how taking action now will help them in future.

Examples of related problems identified in the primary data

- Patients do not know if PR will actually help them.
- The patient believes that PR will not help them – they believe they are too ill to benefit, not bad enough to need it or they feel they do not need it for what they want to do.
- Cannot see that PR will do anything.
- Patients cannot remember what they were told about PR.
- The referrer did not know enough about PR to be able to explain it to the patient.

Examples of what 'good' would look like if the user requirement were satisfied

- Patients have information that helps them understand the difference PR can make to them personally, not just the generic evidence-based benefits of PR.

Requirement 4: Help the patient to feel reassured about any anxieties they have about PR

User needs that the user requirement will address

- Patients need interactions and information that are supportive and encouraging and address their personal anxieties.
- Information needs to be provided in a way the patient can relate to (e.g., vulnerable people lose paper; good relationships and reassurance are important for the frightened).

Examples of related problems identified in the primary data

- Patients are afraid of exercise and becoming short of breath.
- In some cultures, exercise is not something that is encouraged.
- Patients are anxious about going into new situations.
- Patients are embarrassed and self-conscious about their condition.
- PR seems daunting to people who feel unwell.
- Patients experience depression and low morale.
- Patients think they will come under pressure to quit smoking.
- Patients may receive little explanation of PR.
- There may be little shared decision making. "My referral was one sentence, I think you ought to go to pulmonary rehab"
- Patient is worried they will not be able to take someone with them.

Examples of what 'good' would look like if the user requirement were satisfied

- Patients have an opportunity to discuss their fears or anxieties during the referral process.
- Patients believe that in going to PR they will have the whole of the PR team behind them.
- Patients feel that PR will be a welcoming place.

Requirement 5: Help the HCP to understand what happens on a PR programme

User needs that the user requirement will address

- There needs to be awareness raising and training for practice nurses and GPs.
- HCPs need clear, easy to access information about what happens on a PR programme.

Examples of related problems identified in the primary data

- HCPs do not have a clear understanding of what happens on a PR programme.
- HCPs have not visited a PR programme to see first-hand what happens.
- HCPs have not had education about PR from PR providers.

Examples of what 'good' would look like if the user requirement were satisfied

- HCPs have a clear enough understanding of what happens on a PR programme to enable them to feel confident in explaining it to the patient and relating it to the patient's needs.

Requirement 6: Help the HCP to understand the benefits that patients can gain from PR

User needs that the user requirement will address

- HCPs need training on benefits of PR, who can benefit, who to refer and when to refer.
- HCPs need to understand the value of PR relative to other treatments, e.g. how PR can intervene in a vicious circle – cannot breathe, scared, do not go out.

Examples of related problems identified in the primary data

- PR was not part of respiratory nurse training.
- Some HCPs look at PR as a last resort, rather than an integral part of treatment.
- Referral may be less likely if the HCP does not have a special interest in respiratory conditions.
- "Referral to PR comes from having a special interest in respiratory conditions. Nurses that are doing COPD review and just ticking the boxes won't even be thinking about pulmonary rehab, because even if it's on the template if you're not aware of the service you're not going to be telling people about it."
- "I know the doctors wouldn't talk about pulmonary rehab with the patients, they, it's just not in their psyche of where can I send this patient to make him better"

Examples of what 'good' would look like if the user requirement were satisfied

- HCPs learning about PR first hand from patients.
- HCPs are aware of the evidence for PR.
- HCPs understand the triggers that suggest PR would help – e.g. struggling to do every day activities, deteriorating quality of life.
- Referrer knows what types of patients benefit and at what stage of their disease.

Requirement 7: Help the HCP to feel positive about PR and value it as a treatment

User needs that the user requirement will address

- There is a need for HCPs to positively value PR as a treatment for COPD and appreciate its role in the overall management of COPD

Examples of related problems identified in the primary data

- There is pressure from so many clinical priorities in primary care, it might not be the first thing on the HCP's agenda
- A doctor or nurse might not think of PR if they don't have an interest in COPD
- Acute respiratory nurses in the community are not setting an example by referring patients themselves
- HCPs might not see the long term cost value of PR.

Examples of what 'good' would look like if the user requirement were satisfied

- PR is regarded as an essential part of treatment for COPD.
- HCPs refer newly diagnosed patients, those who attended PR more than 1 year ago or who have had exacerbation of symptoms whilst on maximum inhaler therapy.
- HCPs are suggesting PR at every annual review.
- Referrers attend study days where PR providers present.
- HCPs are involved in professional interest groups, e.g. FORD (Focus on Respiratory Disease) organised by Primary Care Respiratory Society.
- The CCG identifies PR champions and links practices together
- HCPs encourage patients who have attended a course to go back and talk about the course, promote it to their GPs and nurses.

Requirement 8: Help HCPs, patients and carers to feel they have had a positive conversation about PR

User needs that the user requirement will address

- Training for HCPs on how to explain PR to the patient.
- Motivational interviewing strategies.
- Detailed information for HCPs to share with patients about what is involved in PR.

Examples of related problems identified in the primary data

- The referring clinician does not understand what happens in PR and so/or finds it difficult to ‘pitch’ PR to patients.
- HCPs may struggle to support patients to take ownership of their condition.
- No shared decision-making tool available.
- If there is no continuity of care between the HCP and the patient, it can make the PR conversation harder.
- “Dr didn’t mention much about it, he said it would show the ways of bringing your phlegm up and everything.” (Patient who declined PR)

Examples of what ‘good’ would look like if the user requirement were satisfied

- HCPs are enthusiastic and knowledgeable about PR.
- HCPs can give examples of similar patients who have been helped.
- Patient receives a recommendation to attend from a trusted clinician.
- Patient believes that PR will improve their health / function / symptoms and is motivated to improve their health.
- Quality of care is emphasised over QOF. A patient focussed approach.
- PR is part of the care planning process and patients are encouraged to be partners in their care.
- HCP has effective materials to aid the conversation.
- If patients decline PR, they are told they can change their mind later.

Supplementary Notes 2:

Barriers and enablers to PR referral and uptake that were out of scope for the current study[13]

Barriers

- Patient has too many other life commitments
- Reliance of family for transport (South Asian patients)
- Communication clarity may be harder to achieve where non-professional interpreters are involved
- HCP is unsure of referral criteria
- Lack of available referral protocols
- Lack of GP support for nurse referral
- HCPs under time pressure
- Lack of continuity of care from GPs
- Lack of data sharing among GP practices

Enablers

- Patient can get to the class
- High value placed on healthcare advice (South Asian patients)
- Face-to-face conversations about PR, not relying on leaflets
- Discussions about PR take place repeatedly over the course of several consultations
- Nurse-led referral process
- System prompts HCP to offer/refer PR
- Administrative support for referral
- HCP has specialist respiratory skills

Supplementary Table 1:

Breakdown of raw votes by each group for each requirement: votes cast before group discussion

	Votes for *Essential*			TOTAL VOTES: ESSENTIAL	Votes for *Desirable*			TOTAL VOTES: DESIRABLE
	Group 1 n=6	Group 2 n=5	Group 3 n=7		Group 1 n=6	Group 2 N=5	Group 3 N=7	
**Requirement 1**	6	5	7	**18**	0	0	0	**0**
**Requirement 2**	6	4	7	**17**	0	1	0	**1**
**Requirement 3**	3	3	6	**12**	3	2	1	**6**
**Requirement 4**	6	3	6	**15**	0	2	1	**3**
**Requirement 5**	6	5	6	**17**	0	0	1	**1**
**Requirement 6**	6	4	6	**16**	0	1	1	**2**
**Requirement 7**	5	4	7	**16**	1	1	0	**2**
**Requirement 8**	6	5	6	**17**	0	0	1	**1**

Supplementary Table 2:

Breakdown of raw votes by each group for each requirement: votes cast after group discussion

	Votes for *Essential*			TOTAL VOTES: ESSENTIAL	Votes for *Desirable*			TOTAL VOTES: DESIRABLE
	Group 1 N=6	Group 2 N=5	Group 3 N=7		Group 1 N=6	Group 2 N=5	Group 3 N=7	
**Requirement 1**	6	5	7	**18**	0	0	0	**0**
**Requirement 2**	6	5	7	**18**	0	0	0	**0**
**Requirement 3**	4	5	5	**14**	2	0	2	**4**
**Requirement 4**	6	3	6	**15**	0	2	1	**3**
**Requirement 5**	6	5	6	**17**	0	0	1	**1**
**Requirement 6**	6	4	5	**15**	0	1	2	**3**
**Requirement 7**	6	5	7	**18**	0	0	0	**0**
**Requirement 8**	6	5	7	**18**	0	0	0	**0**

Supplementary Table 3:

Aggregated score values after two rounds of voting

	Score 0-180	Ranking
**Requirement 1**	162	1
**Requirement 2**	157	2
**Requirement 3**	104	8
**Requirement 4**	126	7
**Requirement 5**	150	5
**Requirement 6**	131	6
**Requirement 7**	152	4
**Requirement 8**	157	2
